# Prune Belly Syndrome in a Female Newborn following In Vitro Fertilization-Induced Pregnancy: A Case Report and Literature Review

**DOI:** 10.1155/2023/5521590

**Published:** 2023-11-29

**Authors:** Ibraheem M. Alkhawaldeh, Jaber H. Jaradat, Mohammad Al-Jafari, Abdulqadir J. Nashwan, Samer Irshaid Alrahamneh

**Affiliations:** ^1^School of Medicine, Mutah University, Al-Karak, Jordan; ^2^Hamad Medical Corporation, Doha, Qatar; ^3^Al-Bashir Hospital, Ministry of Health, Amman, Jordan

## Abstract

Prune belly syndrome (PBS) is a rare congenital anomaly characterized by a triad of abdominal flaccidity, varying degrees of urinary system involvement, and cryptorchidism. The exact cause of PBS is unknown. Clinical symptoms can range from stillbirth to significant renal and respiratory abnormalities to almost normal children. Treatment typically involves surgical repair of the abdominal wall defect and urinary tract abnormalities, early orchiopexy, and supportive management of related problems. We report the first case of a female newborn with PBS following in vitro fertilization-induced pregnancy with a comprehensive systematic review of all relevant cases.

## 1. Introduction

Prune belly syndrome (PBS) is distinguished by the triad of lax “prune-like” abdominal wall secondary to deficient or missing abdominal wall skeletal musculature, urinary tract distension from dysfunctional smooth muscle or ectasia of the urinary system, and bilateral intraabdominal testes [1, 2]. Approximately 1 in 29,000-40,000 live births are affected, with 95% occurring in males [2, 3]. Rarely, female patients with PBS have deficiencies in the abdominal wall and urinary system abnormalities without any gonadal anomalies [2]. Despite advances in the care of children with PBS, this condition continues to be associated with high perinatal mortality, which is likely related to associated prematurity, pulmonary complications, and urinary tract malformations [1, 2]. Here, we present the first case of prune belly syndrome in an in vitro fertilization (IVF) female patient and a systematic review of 24 similar cases.

## 2. Case Presentation

A female infant born at the 38th week of gestation with a 2 kg birth weight was delivered via cesarean section. The patient was not born to a consanguineous marriage and the mother's age was 21 with gravida one para one. IVF was indicated due to the husband's low sperm count. The couple had four failed IVF attempts before they finally achieved a successful pregnancy. No workup was performed after the failed attempts to know the cause, as no pregnancy was observed, and the implantation was poor. Also, no preimplantation genetic diagnosis was carried out because the parents could not afford it and they approved proceeding without it. Fetus sex was vague at 15 weeks of gestation and was confirmed by chromosomal analysis for amniotic fluid to be (46, XX), and it revealed neither intra- or inter-chromosomal abnormality nor mosaicism. However, this test does not detect subtle or submicroscopic rearrangements, low-level mosaicism, or maternal cell contamination.

**Figure 1:**
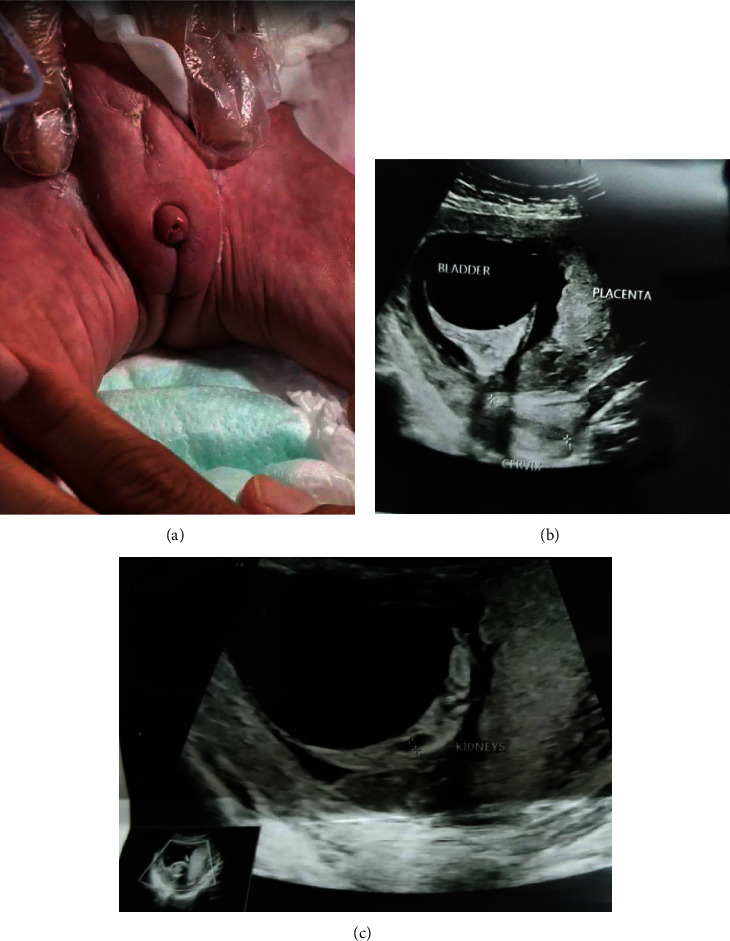
(a) The baby a few hours after birth. During bladder tap. Ambiguous genitalia can be observed. (b) Enlarged bladder extending to the middle of the abdomen. (c) A pea-shaped left kidney expanded by urine recuring from the bladder (from right to left).

Prenatal ultrasonography (US) revealed a single viable fetus with decreased amniotic fluid volume, membrane separation, and cord cyst. Large mega-cystitis was seen occupying the pelvis at the site of the ureter and bladder (UB), extending to the middle of the abdomen, revealing a severely distended UB containing 150 mL echogenic turbid fluid. The urinary outlet was completely obstructed, and the urine was recuring back to the kidneys, leading to kidney expansion and forming a characteristic pea kidney shape. The intestines were dilated with mostly meconium and wedge-shaped (2.5 cm) without ascites. The left kidney appeared echogenic (41 × 19 mm), showing moderate hydronephrosis and ipsilateral hydroureter formation. Although hydronephrosis was observed, corticomedullary differentiation was preserved. The right kidney was not clearly visualized and was seen as an echogenic structure measuring 20 × 9 mm (Figure 1). At this point, a termination of pregnancy was offered to the couple, but they refused due to religious reasons, the age of the fetus, and concerns about the safety of the mother.

At birth, the infant has a prune-like appearance of her abdominal wall muscles, ambiguous genitalia, patent urachus, and an imperforated anus (Figure 1). She was active and not distressed, with stable vitals and a heart rate of 121 beats/min, blood pressure of 67/36 mmHg with normal heart sounds, and O2 saturation of 100%. The head had a wide anterior fontanelle without dysmorphic features and spinal cord scoliosis.

After that, the patient underwent a laparotomy. She was placed in a supine position, and incisions were made for the laparotomy. During the operation, several findings were observed, including hydrometrocolpos, a patent urachus, malrotation of the intestines, internal cloaca (rectum, vagina, and urethra connected to each other), an imperforate anus, and a fistula between the uterus and colon.

The surgical team performed Ladd's procedure and rearranged the small bowel into the right abdomen and the large bowel into the left abdomen. Care was taken to avoid any injury to the intestines during the correction of the malrotation.

Additionally, it was noted that the uterus was connected to the right lower abdomen, and a double bubble colostomy was done; during that, the surgical team carefully assessed the area for any bleeding vessels. They also performed catheterization of the urachus and uterus using the insertion of a Foley's catheter to keep patients. Finally, hydrometrocolpos drains to the right lower abdomen.

**Figure 2:**
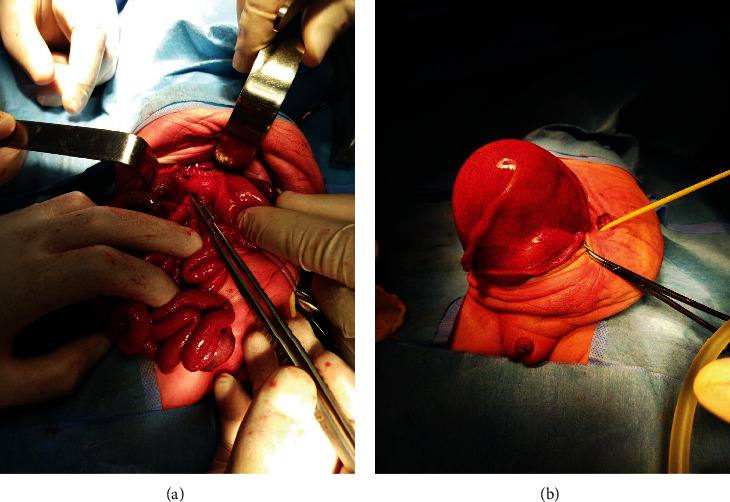
(a) Hydrometrocolpos and (b) small intestine obstruction (from the right to left).

In the following period, she had a series of admissions as a case of upper urinary tract infection, and at 11 weeks old, she was referred to our hospital for having yellowish vomiting for the past day. Moreover, she had passed a small amount of stool in the past 2 days, a history of mild-grade fever (38°C), and abdominal distension in the last 2 days. She looked ill, dehydrated, not distressed, and had a chest good for air entry bilaterally. The abdomen was moving with respiration, moderately distressed, and scarred on the anterior abdominal wall. She was diagnosed with intestinal obstruction necessitating another surgery (Figure 2). The postoperative findings were small bowel obstruction by an adhesion band approximately 100 cm from the duodenojejunal junction and approximately 70 cm from the ileocecal valve, causing proximal small bowel dilation and distal collapse. Laboratory tests were requested; a complete blood count (CBC) assay showed lower hemoglobin level (g/dL9.5), PCV level (31%), and RBC count (3.49 × 106). RDW was 18.3%, and the platelets count was 539 × 103/*u*L. Chemical analysis of the urine showed a turbid yellow color, pH of 5, and negative for proteins and ketone bodies; however, it was nitrite-positive. Microscopically, it was negative for blood (0-2 RBCs) and bilirubin; however, few epithelial cells, 10-12 pus cells, and bacteria were observed. She was given metronidazole injection two times during six days of postoperative follow-up. She was stable and discharged on the sixth day of admission.

**Figure 3:**
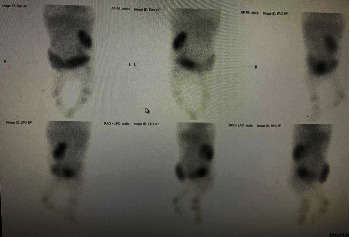
DMSA renal scan.

At 6 months, a 99 m dimercaptosuccinic acid technetium (DMSA) renal scan was performed approximately 2 hours after the DMSA injection to obtain anterior, posterior, and oblique static images. It showed an enlarged left single kidney with a dilated pelvicalyceal system (PCS), mildly hydronephrotic, possibly malrotation, and reduced radiotracer uptake function, especially in the middle cortical region. However, the right kidney was not clearly visualized, suggesting the presence of an absent or nonfunctioning kidney. She was diagnosed with prune belly syndrome with a poor prognosis (Figure 3).

## 3. Systematic Review

### 3.1. Methods

This review was performed and reported according to the meta-analysis (PRISMA) guidelines [4].

### 3.2. Literature Search Strategy

We systematically searched the PubMed, Scopus, and Google Scholar databases. The following search term was used for database search: ((Prune belly syndrome) OR (Eagle Barrett syndrome) OR (Eagle Barrett syndrome) OR (Abdominal Muscle Deficiency Syndrome) OR (Prune-Belly Syndrome) OR (Congenital Absence of the Abdominal Muscles) AND (Female). The last literature search was conducted on February 12, 2023.

### 3.3. Eligibility Criteria and Study Selection

**Figure 4:**
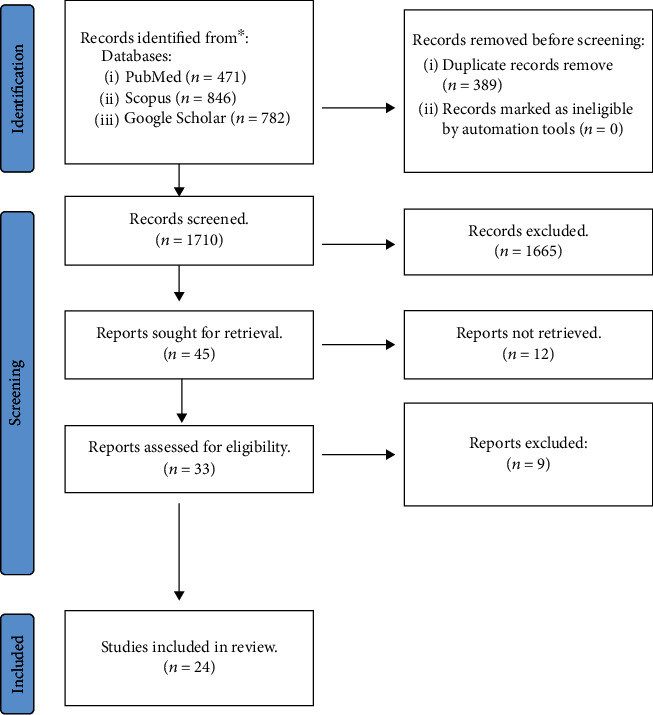
PRISMA flow chart.

Included studies in our systematic review must contain original data on PBS in females. In addition, because the data available on this topic are rare, we decided to include case reports, case series, and letters to the editor. Two independent researchers performed study screening and selection (Figure 4).

## 4. Results

Of the 2099 studies screened, only 24 met the eligibility criteria for this systematic review. Demographic characteristics and clinical data are shown in Table 1. There were nine studies reported from the USA, five from Japan, one from each of the other countries, and two studies did not report the country. The mothers' ages ranged from 19 to 39 years, with a mean age of 27.7 and median age of 28 years. The birth weights (BW) of the babies were between 1520 g and 3560 g, with a mean of 2690 g.

## 5. Discussion

Although Osler introduced the phrase “prune belly syndrome,” Frolich first described it in 1839. In 1950, Eagle and Barrett reported nine cases, who described the condition as the Eagle-Barrett syndrome. Triad syndrome and abdominal musculature deficient syndrome are two other terms reported in the literature [1]. The phrase “prune belly” refers to the distinctively wrinkled appearance of the abdominal wall in newborns caused by a complete or partial lack of abdominal wall muscles [29]. PBS is a diverse congenital disorder with a wide range of clinical manifestations and severities. The clinical manifestations range from stillbirth, primarily caused by significant renal and respiratory dysplasia, to an almost normal infant [1, 29]. Its prognosis depends on the severity of lung and renal dysfunction, and its etiology has not yet been determined [2, 29]. Rare female patients (1.1 per 100,000 [1]) have abdominal wall deficiencies and urinary system abnormalities without any gonadal anomalies [2].

Diagnosis of the syndrome should be considered during prenatal care with an inclusive examination and continuous prenatal follow-up [29]. Therefore, Woodard came up with a classification system for PBS based on prenatal and anatomical characteristics, with category 1 accounting for 20% of children born with PBS. Roughly, it comprises all neonates who die during the first few days of life due to acute renal failure and pulmonary hypoplasia. Additionally, this type is distinguished by oligohydramnios, urethral blockage, patent urachus, or club feet. Patients in category 2 (40%) showed typical PBS symptoms, and their prognosis depends on the degree of renal dysplasia. It is also characterized by hydroureteronephrosis, uropathy, kidney dysplasia, or the risk of urosepsis and azotemia. Patients in category 3 (40%) have modest uropathy, normal renal function, and few PBS clinical characteristics [1, 2]

PBS associations include pulmonary hypoplasia (58%), cardiovascular (25%), gastrointestinal (24%), and musculoskeletal (23%) associations. The imperforate anus was observed in our case and in [5, 6, 14, 17] and [1]. However, Lopes et al. state musculoskeletal association of (30%-45%) comes after the genitourinary tract and abdominal wall abnormalities. With frequent dimpling of the fibular side of the knees, talipes equinovarus (26%), hip dysplasia (5%), and congenital scoliosis (4%) [2], we report congenital scoliosis and dislocated hip in our patient. Two congenital scoliosis were reported in studies [14, 20] and 4 cases with dislocated hips [5, 10, 13, 20]. The probable underlying cause for these abnormalities is the compressive effects of oligohydramnios [2]. Oligohydramnios indicates low urine output, poor renal function, and subsequently hypoplastic lung [1]. However, Lubinsky et al. and Hirose et al. reported polyhydramnios from the late second trimester until about weeks before delivery and normal amniotic fluid, respectively. The patients were free of the above abnormalities supporting the suggested underlying cause [9, 16].

Most cases of PBS are sporadic and have normal karyotypes. However, several studies have identified the hereditary component of PBS [1, 2]. The strongest evidence has been found in several papers that have reported multiplex families with two or more PBS cases [2]. In the past five to ten years, 14 genes have been identified as essential for normal embryonic bladder development and are responsible for the development of a mega-bladder [2]. Al Harbi et al. reported the first female Down syndrome with female PBS; the unusual severity led them to suggest the presence of a modifier gene on chromosome 21 [18]; however, there are not enough cases to associate the trisomy genes (13, 18, and 21) with PBS [1]. PBS was also reported in a Turner syndrome patient by Lubinsky et al.; also, it was severe, and the patient died after 11 days [8]. In our case, there were no chromosomal abnormalities; however, the techniques used do not routinely detect subtle or submicroscopic changes.

PBS is more common in twin pregnancies, whether they are monozygotic or dizygotic. Interestingly, PBS has been recorded in cases of monozygotic twins in both concordance and discordance, suggesting that inherited genetic changes alone cannot explain the pathogenesis of PBS [1]. Although most published twin cases have been discordant for PBS, there have been rare concordant twins with PBS [1]. Two discordant twins were found in the literature, one with PBS and another one healthy [6, 9].

PBS manifests antenatally by the US with features common to bladder outlet blockage, as in dorsal urethral valves or megacystis-megaureter syndrome [2]. A US must reveal a dilated thin-walled bladder, bilateral hydroureters, hydronephrosis, and oligohydramnios to diagnose PBS [1]. We and Morgan et al. [5] observed intra-abdominal calcification. The urachal pseudodiverticulum is usually 2-8 times its normal size and present at birth in 25%-30% of cases like in our case with patent urachus. [2]

PBS patients require a multidisciplinary healthcare approach to aid these children to thrive, gain weight, and be prepared for urological surgery if needed. Orthopedic and psychiatric evaluation and treatment might be necessary for older children. Therefore, individualization of care is recommended, because some patients require abdominal and urinary tract reconstruction while others require as little as bilateral orchiopexies. Up to 40% of patients, particularly those with impaired renal function at initial evaluation, develop chronic renal failure during childhood or adolescence [2]. Perinatal mortality rates for PBS are between 10% and 25%. This is primarily related to the degree of pulmonary hypoplasia, comorbid conditions, and prematurity [1]. Four patients died within the first day [5, 6, 10, 27] and 7 patients in the first month [5, 6, 8, 10, 13, 18, 27]; however, our patient and 8 other patients were alive at the time of reporting [12–14, 17, 19, 21–23]. To our knowledge, this is the first case of PBS in a female patient after IVF.

## 6. Conclusion

PBS is a rare congenital disorder that has neither known prevention other than the routine use of screening for fetal anomalies nor specific etiology. Routine antenatal care with the US will help in detecting renal anomalies early and individualized optimal treatment provided to avoid the fatal course of PBS. We report the first case of PBS in an IVF baby.

## Figures and Tables

**Table 1:** Summary of the reported cases.

Study ID	Country	Study design	Publication month and year	*N* (all)	Mother's age (year)	Gravida Para	Delivery time (weeks)	Delivery complications	Birth weight
Morgan et al. [5]	USA	Case report	1978 Apr	1 (2)	24	G2P2	Delivery at 36 weeks and a half (C-section^∗∗^)	None	2500 g
Alford et al. [6]	USA	Case report	1978 Nov	1 (3)	NR^∗^	Primipara	NR	NR	2340 g
Oesch and Hutchins [7]	USA	Case report	1980 Apr	1 (1)	NR	NR	Delivery at 34 weeks (spontaneous)	None	2310 g
Lubinsky et al. [8]	USA	Case report	1980 Dec	1 (2)	21	G2P1	Term delivery (NR)	None	1950 g
Lubinsky and Rapoport [9]	NR	Case report	1983 Feb	1 (2)	26	NR	Delivery at 34 weeks and a half (C-section)	Polyhydramnios and 3 episodes of bleeding	1520 g
Grosse Hokamp and Müller [10])	Germany	Case report	1983 Jun	1 (1)	NR	G2P2	Delivery at 36^th^ week and a half (C-section)	None	1570 g
Nakayama et al. [11]	NR	Case report	1984 Sep	1 (3)	33	NR	Induced delivery at 34^th^ week (NR)	Progressive ascites	2340 g
Ramos et al. [12]	Philadelphia	Case report	1992 Dec	1 (1)	21	G2P0	Delivery at 39^th^ week (breech vaginal)	None	2970 g
Donnelly and Johnson [13]	USA	Case report	1995 Apr	2 (2)	Case 1: 39 Case 2: NR	Case 1: G2P1 Case 2: NR	Case 1: delivery at 38^th^ week (NR) Case 2: delivery at 36^th^ week (NR)	Case 1: gestational diabetes Case 2: preterm labor	Case 1: 3530 g Case 2: 2450 g
Yoshida et al. [14]	Japan	Case report	1995	1 (1)	27	G1P1	Delivery at 38^th^ week (NR)	NR	2818 g
Güvenç et al. [15]	Turkey	Case report	1995 Jun	1 (1)	19	G1P1	Delivery at 39^th^ week (NR)	NR	2920 g
Hirose et al. [16]	Japan	Case report	1995 Sep	1 (1)	30	G3P2	Delivery at 38^th^ week (C-section)	Premature rupture of the membrane	2880 g
Kanamori et al. [17]	Japan	Case report	2001 May	1 (1)	NR	NR	Delivery at 35^th^ week and 6 days (C-section)	NR	NR
Al Harbi [18]	Saudi Arabia	Case report	2003 Nov	1 (1)	32	G8P6+1	Term delivery (C-section)	NR	3060 g
Bogart et al. [19]	USA	Case report	2006 Jul	1 (2)	20	NR	Delivery at 29^th^ week (spontaneous vaginal)	Oligo-hydramnios	NR
Ely et al. [20]	USA	Case report	2008 Jul	1 (1)	NR	NR	Delivery at 31 weeks (C-section)	Gestational diabetes	
Giuliani et al. [21]	USA	Case report	2010 Nov	1 (1)	29	G2P1	Delivery at 36^th^ week and 4 days (C-section)	Spontaneous rupture of membrane	3560 g
Oka et al. [22]	Japan	Case report	2011 Oct	1 (1)	NR	NR	NR	NR	NR
Hillman et al. [23]	USA	Case report	2012 Nov	1 (1)	NR	NR	Term delivery (vaginal)	PBS	3430 g
Samal and Rathod [24]	India	Case report	2015 Jan	1 (1)	39	G5P5	Term delivery (NR)	NR	2700 g
Travan et al. [25]	Italy	Case report	2016 Jul	1 (1)	29	G2P1	Term delivery (vaginal)	Fetal hydrops and anemia	3058 g
Wijesinghe et al. [26]	New Zeland	Case report	2016 Aug	1 (1)	38	primipara	Delivery at 31 weeks (NR)	DM-1, hypertension, and preeclamptic toxemia	NR
Peña-Padilla et al. [27]	Mexico	Case report	2019 Oct	1 (1)	NR	NR	Delivery at 44^th^ week (C-section)	NR	3200 g
Inaguma et al. [28]	Japan	Case report	2020 Nov	1 (1)	NR	NR	NR	NR	NR

^∗^NR: not reported. ^∗∗^C-section: cesarean section.

## Data Availability

All data generated or analyzed during this study are included in this published article.

## References

[1] Hassett S., Smith G. H. H., Holland A. J. A. (2012). Prune belly syndrome. *Pediatric Surgery International*.

[2] Lopes R. I., Baker L. A., Dénes F. T. (2021). Modern management of and update on prune belly syndrome. *Journal of Pediatric Urology*.

[3] Rabinowitz R., Schillinger J. F. (1977). Prune belly syndrome in the female subject. *The Journal of Urology*.

[4] Page M. J., McKenzie J. E., Bossuyt P. M. (2021). The PRISMA 2020 statement: an updated guideline for reporting systematic reviews. *BMJ*.

[5] Morgan C. L., Grossman H., Novak R. (1978). Imperforate anus and colon calcification in association with the prune belly syndrome. *Pediatric Radiology*.

[6] Alford B. A., Peoples W. M., Resnick J. S., L’Heureux P. R. (1978). Pulmonary complications associated with the prune-belly syndrome. *Radiology*.

[7] Oesch I., Hutchins G. M. (1980). Prune belly syndrome in a female with presentation simulating diaphragmatic hernia1. *European Journal of Pediatric Surgery*.

[8] Lubinsky M., Doyle K., Trunca C. (1980). The association of “prune belly” with Turner’s syndrome. *American Journal of Diseases of Children*.

[9] Lubinsky M., Rapoport P. (1983). Transient fetal hydrops and prune belly in one identical female twin. *New England Journal of Medicine*.

[10] Grosse Hokamp H., Müller K. M. (1983). Prune belly syndrome and female pseudohermaphroditism. *Pathology Research and Practice*.

[11] Nakayama D. K., Harrison M. R., Chinn D. H., Lorimier A. A. (1984). The pathogenesis of prune belly. *American Journal of Diseases of Children*.

[12] Ramos F. J., McDonald-McGinn D. M., Emanuel B. S., Zackai E. H. (1992). Tricho-rhino-phalangeal syndrome type II (Langer-Giedion) with persistent cloaca and prune belly sequence in a girl with 8q interstitial deletion. *American Journal of Medical Genetics*.

[13] Donnelly L. F., Johnson J. F. (1995). Unilateral abdominal wall hypoplasia: radiographic findings in two infant girls. *Pediatric Radiology*.

[14] Yoshida M., Matsumura M., Shintaku Y. (1995). Prenatally diagnosed female prune belly syndrome associated with tetralogy of fallot. *Gynecologic and Obstetric Investigation*.

[15] Güvenç M., Güvenç H., Aygün A. D., Yalçin O., Baydinç Y. C., Soylu F. (1995). Prune-belly syndrome associated with omphalocele in a female newborn. *Journal of Pediatric Surgery*.

[16] Hirose R., Suita S., Taguchi T. (1995). Prune-belly syndrome in a female, complicated by intestinal malrotation after successful antenatal treatment of hydrops fetalis. *Journal of Pediatric Surgery*.

[17] Kanamori Y., Hashizume K., Kawarasaki H., Kitano Y., Sugiyama M., Tanaka Y. (2001). Single vaginal ectopic ureter and renal hypoplasia associated with urogenital sinus and abdominal muscular hypoplasia--a novel subtype of prune-belly syndrome in a female child?. *Urology*.

[18] Al Harbi N. N. (2003). Prune-belly anomalies in a girl with down syndrome. *Pediatric Nephrology*.

[19] Bogart M. M., Arnold H. E., Greer K. E. (2006). Prune-belly syndrome in two children and review of the literature. *Pediatric Dermatology*.

[20] Ely B., Gustafson R. A., Karnsakul W. (2008). Pseudoprune-belly syndrome in vertebral abnormalities, anal atresia, cardiac abnormalities, tracheoesophageal fistula and/or esophageal atresia, renal agenesis and dysplasia, and limb defects association. *Clinical Gastroenterology and Hepatology*.

[21] Giuliani S., Vendryes C., Malhotra A., Shaul D. B., Anselmo D. M. (2010). Prune belly syndrome associated with cloacal anomaly, patent urachal remnant, and omphalocele in a female infant. *Journal of Pediatric Surgery*.

[22] Oka Y., Masumoto K., Nakamura M., Iwasaki A. (2011). Colonic volvulus detected by CT scan in a case with mental retardation and prune belly syndrome. *Asian Journal of Surgery*.

[23] Hillman R. T., Garabedian M. J., Wallerstein R. J. (2012). Pregnancy outcome in a woman with prune belly syndrome. *BMJ Case Reports*.

[24] Samal S. K., Rathod S. (2015). Prune belly syndrome: a rare case report. *Journal of Natural Science, Biology, and Medicine*.

[25] Travan L., Naviglio S., Cont G., Brovedani P., Davanzo R., Demarini S. (2016). Isolated hypoplasia of abdominal wall muscles associated with fetal ascites. *Congenital Anomalies*.

[26] Wijesinghe U. S., Muthucumaru M., Beasley S. W. (2016). Further evidence of the etiology of prune belly syndrome provided by a transient massive intraabdominal cyst in a female. *Journal of Pediatric Surgery*.

[27] Peña-Padilla C., Viramontes-Aguilar L., Tavares-Macías G. (2019). Pfeiffer syndrome type 3 and prune belly anomaly in a female: case report and review. *Fetal and Pediatric Pathology*.

[28] Inaguma Y., Kaito H., Tanaka R. (2020). A rare case of peritonitis in a young woman on peritoneal dialysis. *CEN Case Reports*.

[29] Achour R., Bennour W., Ksibi I. (2018). Prune belly syndrome: approaches to its diagnosis and management. *Intractable & Rare Diseases Research*.

[30] Alkhawaldeh I. M., Jaradat J. H., Al-Jafari M., Nashwan A. J., Alrahamneh S. I. First case of female prune belly syndrome following in vitro fertilization-induced pregnancy: a rare case report and systematic review of the literature. https://www.researchsquare.com/article/rs-3061077/v1.

